# Biomedical relation extraction method based on ensemble learning and attention mechanism

**DOI:** 10.1186/s12859-024-05951-y

**Published:** 2024-10-18

**Authors:** Yaxun Jia, Haoyang Wang, Zhu Yuan, Lian Zhu, Zuo-lin Xiang

**Affiliations:** 1 Department of Radiation Oncology, Shanghai East Hospital, Tongji University School of Medicine, Shanghai, China; 2 Department of Computer College, Beijing Information Science and Technology University, Beijing, China; 3 Department of Information Management, The National Police University for Criminal Justice, Baoding, China; 4 Department of Radiation Oncology, Shanghai East Hospital Ji’an hospital, Jian, China

**Keywords:** Biomedical relation extraction, Deep learning, BERT, Stacking, Attention mechanism

## Abstract

**Background:**

Relation extraction (RE) plays a crucial role in biomedical research as it is essential for uncovering complex semantic relationships between entities in textual data. Given the significance of RE in biomedical informatics and the increasing volume of literature, there is an urgent need for advanced computational models capable of accurately and efficiently extracting these relationships on a large scale.

**Results:**

This paper proposes a novel approach, SARE, combining ensemble learning Stacking and attention mechanisms to enhance the performance of biomedical relation extraction. By leveraging multiple pre-trained models, SARE demonstrates improved adaptability and robustness across diverse domains. The attention mechanisms enable the model to capture and utilize key information in the text more accurately. SARE achieved performance improvements of 4.8, 8.7, and 0.8 percentage points on the PPI, DDI, and ChemProt datasets, respectively, compared to the original BERT variant and the domain-specific PubMedBERT model.

**Conclusions:**

SARE offers a promising solution for improving the accuracy and efficiency of relation extraction tasks in biomedical research, facilitating advancements in biomedical informatics. The results suggest that combining ensemble learning with attention mechanisms is effective for extracting complex relationships from biomedical texts. Our code and data are publicly available at: https://github.com/GS233/Biomedical.

## Introduction

**Fig. 1:**
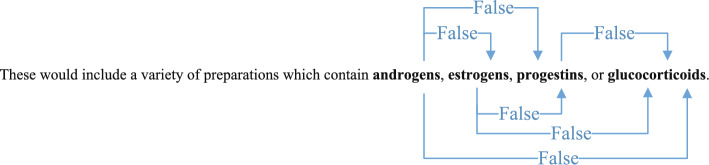
Example of relation extraction in DDI datasets

As the corpus of biomedical literature experiences exponential expansion, the task of manually curating and organizing this vast expanse of information becomes increasingly complex and daunting. In biomedical research [1–4], relation extraction (RE) emerges as a cornerstone task, essential for uncovering and distilling the intricate semantic relationships among entities mentioned within textual data. These relationships encompass protein-protein interactions (PPI) [5], drug-drug interactions (DDI) [6], and chemical-protein interactions (ChemProt)[7], etc. Figure 1 shows an example of relation extraction in DDI datasets. The sentence ’These would include a variety of preparations which contain androgens, estrogens, progestins, or glucocorticoids’ is analyzed for potential relationships between the mentioned drug categories. The arrows indicate the presence or absence of relation between these categories, with ’False’ indicating no relation. By automatically extracting and cataloging relevant interactions from the ever-growing pool of biomedical literature, these systems enable researchers to swiftly identify potential drug targets, predict adverse drug reactions, and elucidate the molecular mechanisms underlying complex diseases. Moreover, the effective extraction of these relationships is critical for advancing our understanding of complex biological systems and plays a pivotal role in facilitating subsequent tasks in automated reasoning, machine translation, and question-answering. Given the significance of RE in the broader landscape of biomedical informatics and the increasing volume of literature that necessitates efficient processing, there is a pressing need for sophisticated computational models that can accurately and efficiently extract these relationships at scale.

Currently, biomedical relationship extraction methods can be broadly categorised into two types: pattern-based methods and machine learning-based methods. Pattern-based methods [8–13] are traditional approaches that employ specific patterns and matching rules to identify semantic relationships between biomedical entities. The effectiveness of these methods largely depends on the quality and quantity of predefined patterns or rules. However, as these patterns or rules often cannot adapt to variations in text expression, pattern-based methods tend to have lower recall rates. Machine learning-based methods can be classified into three categories: feature-based methods, kernel-based methods and neural network-based methods. Feature-based methods [14–17] involve the definition or extraction of various lexical and syntactic features by experts, making these methods highly dependent on expert knowledge. Kernel-based methods rely on kernel functions to effectively compute the similarity between structural data, but designing appropriate kernel functions is often more challenging than selecting or constructing features. The neural network approach, on the other hand, is a data-driven method that can learn latent feature representations directly from labelled training data, without requiring experts to meticulously design patterns, features or kernel functions. As a result, neural network-based methods have become the dominant technique for biomedical relation classification and are gaining increasing attention in the field. Automated relation extraction (RE) systems, driven by deep learning techniques [18–27], have emerged as a burgeoning trend in research.

In recent years, deep learning-based models, particularly BERT (Bidirectional Encoder Representations from Transformers) [28] and its variants, have attained considerable success across various natural language processing tasks. In the biomedical relation extraction domain, models such as BioBERT, BlueBERT, and PubMedBERT have demonstrated formidable performance by being pre-trained on vast corpora of domain-specific texts. However, these models still exhibit certain limitations. Firstly, since the model’s training data comes from different medical corpora, this means that their generalization ability may be limited in different fields or specific application scenarios. Secondly, although these models have made progress in word-level representation, there are still challenges in dealing with long-distance dependencies and complex contexts.

To tackle these challenges, we propose a novel approach - SARE that leverages ensemble learning Stacking and attention mechanisms, enhancing the performance of biomedical relation extraction. Our approach combines the strengths of multiple pre-trained models, leveraging ensemble learning to bolster the model’s adaptability and resilience across diverse domains and application scenarios. Each base classifier is trained on the same data, but as they learn differently, their diverse perspectives contribute to a more comprehensive understanding of the data. These base classifiers generate predictions that are then used as inputs for a final meta-model, which is trained to synthesize these insights into a cohesive output, thus improving overall prediction accuracy. Moreover, attention mechanisms enable the model to dynamically prioritize parts of the text that are more relevant for identifying and extracting relationships, avoiding the distraction of less relevant information. By incorporating attention mechanisms, SARE is better equipped to capture and utilize key information within the text, thereby enhancing the accuracy of relation extraction. Our method not only learns complementary features from different models but also makes more precise predictions by focusing on the most relevant parts of the text. In addition, the model proposed in this study can not only handle relationship extraction for single entity pairs, but also handle complex situations where the input text contains multiple entities and relationships. For each pair of entities, the model independently generates embedded representations and focuses on the most relevant parts of the text through attention mechanisms to extract precise relationships. In summary, this paper contributes in the following ways:

- We propose a new method - SARE - to increase the effectiveness of biomedical relation extraction by applying a stacking-based ensemble learning strategy. This approach integrates the strengths of multiple diverse models, improving accuracy through a refined meta-classifier that synthesizes their predictions.
- We employ attention mechanisms to enable the model to effectively focus on crucial relations between entities. This targeted approach optimizes the extraction process by dynamically prioritizing the most relevant parts of the text, improving both the precision and accuracy of our results.
- We achieve excellent performance on three benchmark datasets of relation extraction tasks. This result demonstrates the robustness and effectiveness of SARE across various complex biomedical texts and scenarios.

The subsequent sections of the paper are structured as follows: Section Background depicts the background of ensemble learning. The related works of biomedical relation extraction and ensemble classification in deep learning are presented in Section  Related work. In Section Biomedical relation extraction, we detail the methods combining ensemble learning Stacking strategy and attention mechanisms. Section Ensemble classification in deep learning details the evaluation experiments and associated discussions. Ultimately, the paper culminates with conclusions provided in Section Methods.

## Background

As the volume of data continues to surge and deep learning technology advances persistently, single models face challenges in handling complex tasks. To address this issue, ensemble learning can improve the model’s overall performance by integrating the advantages of multiple models [29]. The primary ensemble learning methods include Voting, Bagging, Boosting, and Stacking.

**Fig. 2:**
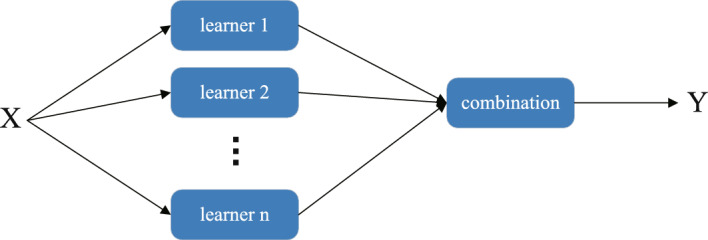
The basic principles of ensemble learning stacking method

In short, Voting improves model performance through collective decision-making, suitable for scenarios where different models perform relatively evenly; Bagging reduces variance by random sampling and averaging, enhancing model stability and generalization ability, ideal for high-variance models; Boosting enhances model performance by sequentially training models and correcting errors, suitable for reducing bias and improving model accuracy; Stacking combines predictions from multiple models and integrates them using a meta-model, offering flexibility to capture the strengths of different models, applicable for scenarios requiring maximal performance enhancement. We have significantly improved the model’s performance by leveraging the Stacking strategy’s advantages. Figure 2 illustrates the basic principles of the ensemble learning stacking method.

## Related work

### Biomedical relation extraction

In this section, we offer a succinct overview of notable works in biomedical relation extraction.

SciBERT [30], designed specifically for scientific text, undergoes unsupervised pre-training on a vast corpus of multi-domain scientific publications. This enhances its performance on various downstream NLP tasks such as sequence tagging, sentence classification, and dependency parsing across a wide range of scientific domains. Experiments show substantial performance gains over BERT across multiple scientific tasks, with SciBERT achieving the optimum performance levels on certain tasks.

BlueBERT [31] is a pre-trained model based on BERT, specifically designed for handling biomedical text data. Pre-trained on an extensive biomedical literature corpus, it gains enhanced comprehension and processing capabilities tailored to the medical domain. The pre-training of BlueBERT utilizes rich biomedical text data, enabling the model to more accurately comprehend medical terminologies, entities, and relationships, aiding in diverse text processing tasks within the biomedical domain.

BioBERT [32], tailored for biomedical tasks, enhances performance in named entity recognition, relation extraction, and question answering tasks through pre-training on large biomedical datasets. It surpasses traditional BERT models, showing superior performance across various biomedical NLP tasks due to its specialization in handling biomedical terminologies and structures.

PubMedBERT [33], in contrast to pre-training on general-domain corpora, undergoes domain-specific pre-training from scratch, resulting in significant performance enhancements. Researchers showcased its effectiveness across diverse biomedical NLP tasks by introducing a comprehensive benchmark, achieving new state-of-the-art results in multiple areas.

Su et al. [34] presents a pioneering contrastive pre-training method that enhances the text representation of BERT models for biomedical relation extraction tasks. This is achieved by integrating linguistic knowledge into data augmentation techniques. It also harnesses external knowledge sources to construct large-scale datasets, enhancing BERT models’ generalization capability through contrastive pre-training. The experimental results demonstrate that this method enhances the representational capacity of BERT models, leading to state-of-the-art performance on three relation extraction benchmark datasets.

Su et al. [35] incorporate sub-domain adaptation during pre-training to align domain and task-specific knowledge. Furthermore, it introduces a novel SSL fine-tuning mechanism that leverages the knowledge from the last layer of BERT to enhance model performance. The goal is to enhance relation extraction tasks by finely tuning the model for biomedical domain requirements.

TreeBERT [36] enhances the performance of relation extraction tasks by amalgamating the formidable capabilities of the BERT model with a tree-like structure, which captures hierarchical relationships within sentences. This method has been tested on multiple biomedical relation datasets and demonstrates its effectiveness in extracting complex relationships between entities, particularly in handling nested entities and multi-level relationships.

K-RET [37] system leverages rich domain knowledge bases and advanced natural language processing techniques to enhance the accuracy and coverage of relation extraction. By integrating pre-trained language models, knowledge graphs, and machine learning algorithms, it demonstrates outstanding performance on biomedical relation extraction tasks. Through the integration of multiple information sources and the adoption of fine-grained relation classification, K-RET provides a powerful tool for the automated mining and organization of biomedical knowledge.

This method [38] represents the first attempt to utilize the BERT model for extracting transcriptional regulatory interactions from biomedical literature. Additionally, it proposes an optimal model based on the LUKE architecture for extracting specific types of regulatory interactions from literature, achieving an accuracy of 82% in reconstructing the Salmonella TRN (Transcriptional Regulatory Network).

Through the analysis of the relevant work on biomedical information extraction, we find that existing methods mostly rely on the BERT model, which significantly enhances the performance of relation extraction. However, differences in the corpora used for model training and their applicability result in a lack of universality and generalization in the models.

### Ensemble classification in deep learning

Ensemble learning methods have demonstrated significant effectiveness in enhancing results across numerous NLP tasks compared to individual deep learning models[39].

Akhtyamova et al. [40] proposed a method based on large-scale convolutional neural network ensembles for predicting drug safety from user comments on health forums. By determining prediction results through voting mechanism, this method significantly improves the accuracy of drug safety predictions, achieving a binary classification accuracy of 87.17% and a multi-class classification accuracy of 62.88%. Araque et al. [41] introduced a sentiment classifier based on voting and stacking. By integrating word embeddings, surface features, and meta-learning techniques, and combining these with various traditional classifiers, the accuracy of sentiment analysis is significantly improved. Akhtar et al. [42] proposed an ensemble model based on voting and stacking that leverages CNN, LSTM, and GRU models along with handcrafted feature representations. By employing a multi-task learning framework to address four problems in sentiment and emotion analysis, the model achieves better results on multiple datasets compared to single-task learning. Heikal et al. [43] combined CNN and LSTM through soft voting, the model achieved a higher F1 score on the Arabic Sentiment Tweets Dataset compared to existing state-of-the-art models. AI-Omari et al. [44] combined BiLSTM, XGBoost, and BERT technologies via voting to detect fake news. In the NLP4IF 2019 shared task, this model performed exceptionally well in sentence-level classification tasks, significantly outperforming the baseline models. Minaee et al. [45] introduced a sentiment analysis framework based on an ensemble model combining CNN and Bi-LSTM. The model leverages CNN to capture local structural information in the data and utilizes Bi-LSTM to extract temporal relationships. By combining the prediction scores of these two models, the framework improves the accuracy of sentiment analysis. Haralabopoulos et al. [46] proposed a baseline model based on stacked and weighted ensembles for multi-label binary classification of user-generated content. Evaluations on two datasets show an average improvement in classification accuracy ranging from 1.5 to 5.4%. Wang et al. [47] introduced a method that uses Particle Swarm Optimization (PSO) to automatically search for and learn the optimal CNN architecture for maximized classification accuracy. Livieris et al. [48] proposed two ensemble prediction models based on Bagging and Boosting strategies. Experimental results indicate that the combination of ensemble learning and Weighted Convolutional Neural Networks (WCNNs) can build efficient and robust classification models. Mohammadi et al. [49] proposed an ensemble deep learning approach that integrates four deep learning models (CNN, LSTM, BiLSTM, and GRU). By using a stacking method and logistic regression as a meta-learner to combine the outputs of these models, this approach is designed for aspect-based sentiment analysis. Liang et al. [50] proposed an ensemble learning framework that combines Bagging and AdaBoost algorithms through a two-stage processing workflow and three enhancement strategies to enhance the classification performance of policy texts. Mohammed et al. [51] combined multiple deep learning models to enhance the accuracy and robustness of text classification through ensemble learning voting and stacking techniques. Zheng et al. [52] utilized the BERT pre-trained language model for text word embeddings and combines heterogeneous base classifiers constructed with TextCNN, DPCNN, TextRNN, and TextRCNN. The model?s generalization ability is enhanced through Stacking ensemble learning, using a SVM as the meta-classifier for training and prediction. Chen et al. [53] proposed an ensemble learning model based on TextCNN, which combines ALBERT, RoBERTa, and DistilBERT to extract textual features and employs voting method to enhance the accuracy and robustness of classification.

Ensemble learning combines the strengths of different models to compensate for individual model shortcomings, significantly enhancing the overall performance of models in classification tasks. Therefore, addressing the issues existing in biomedical relation extraction models through ensemble learning algorithms to integrate the advantages of different models and improve model effectiveness is feasible.

## Methods

### Task description

Given a biomedical text sequence $X={x_1, x_2, \cdots , x_n}$, the task is to classify the relations between biomedical entity pairs $E= {(e_1,e_2),(e_3,e_4),\cdots ,(e_{m-1},e_m)}$ in the text. Here, $e_i$ represents the *i*th entity, and $\frac{m}{2}$ indicates the number of entity pairs. Each entity pair $(e_i,e_j)$ corresponds to a relation label $y_{ij}$, where $y_{ij} \in Y$, and *Y* is the predefined label set for biomedical relation extraction datasets.

### Model framework

**Fig. 3:**
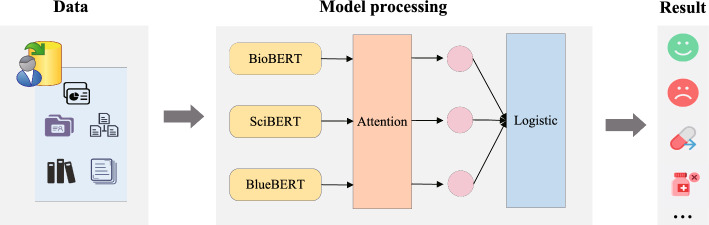
The framework of relation extraction model based on Stacking and attention mechanism

In the biomedical domain, due to the particularity of the relation extraction task, we usually convert it into a classification task. In other words, when provided with a sentence and two specified entities, our objective is to ascertain whether the sentence implies a specific relation between the two entities. But, due to the differences in training data and specific tasks, the model’s performance often varies. Ensemble learning Stacking predicts results by integrating multiple heterogeneous base classifiers, which have stronger scene adaptability and higher classification accuracy. Hence, we introduce a multi-base model framework utilizing the Stacking strategy, which fully takes into account the diversity and learning capabilities among multiple base classifier models. Meanwhile, by integrating the attention weights of different base models, we can more accurately capture key features, thereby enhancing the robustness and generalization ability of the model. In cases where the input text includes multiple entities and relationships, the model processes each entity pair independently. The attention mechanism is employed to focus on relevant parts of the text specific to each entity pair. Following this, the model predicts the relationship for each entity pair separately. The results are then aggregated to form the final output, ensuring that multiple relationships within the same text are accurately represented. This method is effective for managing complex inputs with several entities and relationships. Figure 3 demonstrates the framework of the relation extraction model based on Stacking and attention mechanisms. The entire framework consists of four modules: embedding module, encoding module, ensemble module, and predication module.

### Embedding module

The embedding module in the context of relation extraction tasks refers to the initial stage where input entities and contextual information are encoded into dense vector representations. Mathematically, given a set of input entities $E$ represented as tokens $e_i$ (where $i = 1, 2,..., n$), each entity token is mapped to a fixed-size dense vector $v_i$ through an embedding matrix $W_{emb}$, such that $v_i = W_{emb} \cdot e_i$. These embeddings capture semantic and syntactic information about the entities, enabling the neural network to effectively process and learn from the input data while preserving contextual relationships between entities. Through the training process, the parameters of the embedding matrix $W_{emb}$ are optimized to minimize the loss function, thereby enhancing the network’s ability to extract meaningful relations between entities from the input text.

### Encoding module

This paper applys several typical BERT models in the biomedical field to encode text. Firstly, the basic principle of BERT was introduced, followed by the characteristics of BioBERT, BlueBERT, and PubMedBERT respectively.

#### BERT model

**Fig. 4:**
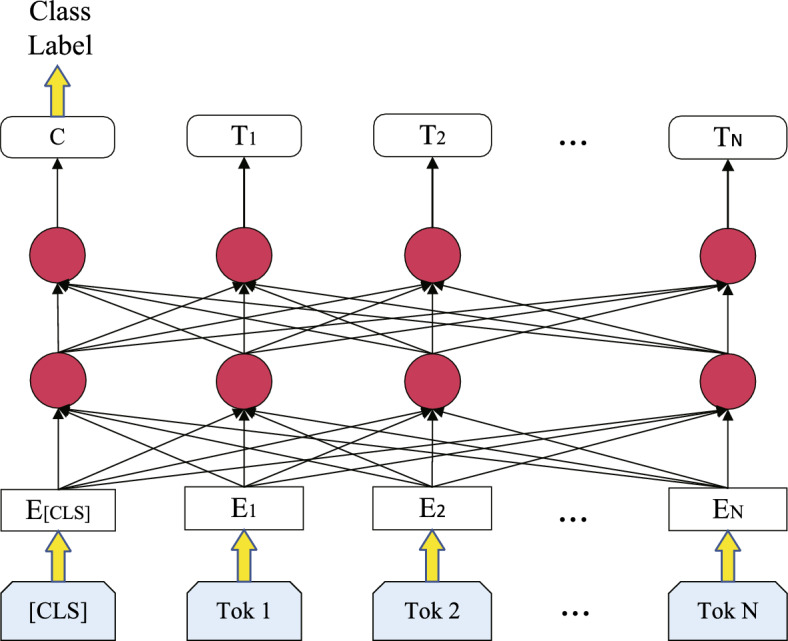
Schematic diagram of BERT model structure

BERT is a Transformer-based pre-trained model. Unlike previous unidirectional language models, BERT can simultaneously consider the left and right context of each word in the text, enabling it to better understand language structure with greater accuracy. Simultaneously, this also enables BERT to capture subtle nuances of vocabulary in different contexts. By conducting pre-training on large-scale corpora, BERT can generate deep and high-quality language representations, showcasing outstanding performance across various NLP tasks including text classification, question answering systems, and semantic understanding. Additionally, BERT’s pre-training and fine-tuning mechanism greatly enhance model flexibility and adaptability, allowing it to swiftly adapt to the specific requirements of particular tasks. Figure 4 is the framework diagram of the BERT model.

The BERT model comprises input representations and semantic extraction as its core components. Input representations typically consist of three parts: token embeddings, segment embeddings, and positional embeddings. The semantic extraction component within the BERT model is a multi-layer bidirectional decoder built upon the Transformer encoder. It consists of three components: multi-head attention mechanism, layer normalization and residual connections, and feed-forward neural network.

#### Base classifier model

Currently, in the field of biomedicine, variants of the BERT model are primarily employed for biomedical relation extraction research. To address the performance discrepancies caused by data training and fine-tuning in individual models, we utilize the three mainstream models - BioBERT, BlueBERT, and PubMedBERT - to construct base classifiers for Stacking-based ensemble learning.

(1) **BioBERT**: is based on the BERT model pre-trained on large-scale biomedical corpora, enabling it to better understand and process complex texts in the biomedical domain. Compared to BERT in general domains, BioBERT demonstrates significant performance improvements in tasks such as biomedical named entity recognition, biomedical relation extraction, and biomedical question answering, thereby enhancing the effectiveness of biomedical text mining. Additionally, BioBERT’s pre-training process involves fine-tuning the model, optimizing it for specific biomedical tasks. In this way, BioBERT not only retains the powerful feature extraction and representation capabilities of the BERT model but also further enhances performance and adaptability in biomedical text processing tasks through domain-specific pre-training.

(2) **BlueBERT**: adopts the Transformer architecture and leverages large-scale clinical medical text data for pre-training. It not only undergoes pre-training on extensive biomedical literature but also leverages various biomedical-related tasks, including named entity recognition, relation extraction, and question answering systems, to further enhance model performance. Moreover, BlueBERT achieves significant performance improvements across different biomedical text mining tasks by integrating weights from multiple pre-trained models using a method called model distillation.

(3) **PubMedBERT**: A pre-trained language model, tailored specifically for biomedical text, constructed upon the BERT architecture. Its core principle involves utilizing a vast PubMed literature corpus for pre-training, thereby enhancing the model’s grasp of the semantics and contexts within the biomedical domain. PubMedBERT owns better adaptability to biomedical domain-specific terms and entities, as well as outstanding performance on tasks such as biomedical relation extraction.

Each of these three models is tailored for different target tasks, each possessing its unique strengths and characteristics. They exhibit varying adaptability across different scenarios and demonstrate robust performance across multiple natural language processing (NLP) tasks.

### Model processing based on stacking and attention

**Fig. 5:**
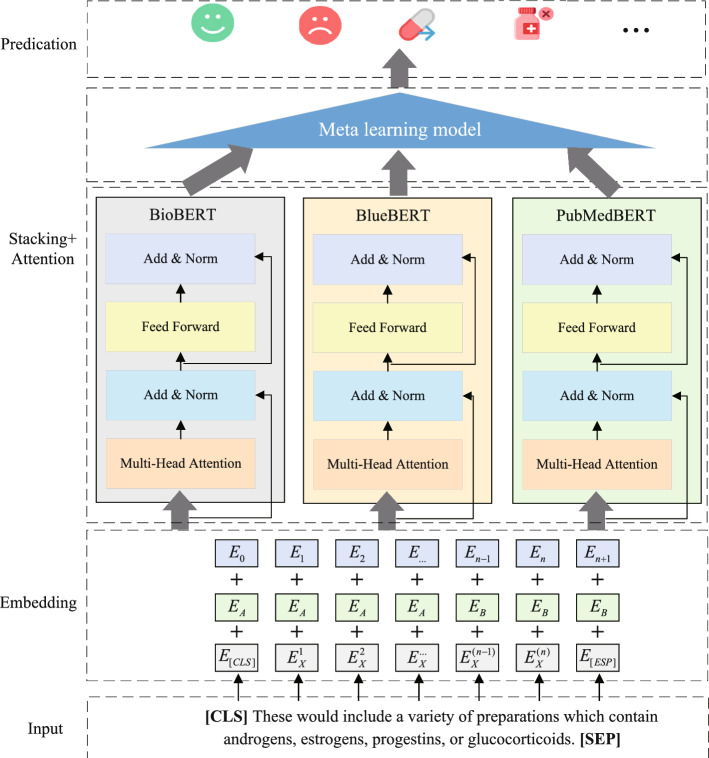
The biomedical relationship extraction detail process based on Stacking and attention

Figure 5 shows in detail the biomedical relationship extraction process based on Stacking and attention. For Stacking, we first establish base models. We have selected three pre-trained models: BioBERT, BlueBERT, and PubMedBERT. These models have been pre-trained in the biomedical domain, thus exhibiting strong performance for tasks in this field. Each of these models is trained on the same dataset but processes the data independently, allowing each model to capture different aspects or features of the biomedical text. After training, each base model generates its predictions for the input data. These predictions include the probability distributions over possible relation labels for each pair of entities in the text. The predictions from the base models are then used as inputs to a meta-model. In our implementation, the meta-model is a logistic regression classifier. This meta-model learns to combine the outputs of the base models by assigning appropriate weights to their predictions. The result is a final prediction that benefits from the strengths of each individual model, thereby improving the overall accuracy and robustness of the system. For each base model, the input comprises text data, and the output corresponds to the classification or labeling of the text. Specifically, suppose there are *N* training samples $(X_i, y_i)$, where $X_i$ is the text input and $y_i$ is the corresponding label. For each base model $M_j$, you can obtain the prediction result $P_j(X_i)$, where $P_j$ is the prediction function of model $M_j$ for input $X_i$. Therefore, for each sample *i* and each base model *j*, you obtain a prediction matrix *P*, where the (*i*, *j*)-th element is the prediction of model $M_j$ for input $X_i$.

Next, we compute the attention weights for the predictions of each base model. The purpose of attention is to allow the model to dynamically focus on the most relevant parts of the input text. This is crucial for accurately identifying relationships in biomedical texts, where certain terms or phrases carry significant importance. Within each base model, attention weights are calculated based on the relevance of different words or phrases in the input sequence. These weights determine the contribution of each part of the text to the final output. By focusing on the most informative segments, the model can more effectively extract the correct relationships. After applying attention, the outputs from the base models (now adjusted by attention weights) are passed to the meta-model, which synthesizes these results into a final decision. This approach ensures that the final prediction is not only a blend of the strengths of multiple models but also a refined output that emphasizes the most critical information in the text. If we employ an attention function to compute the attention weights, for each sample *i* and each base model *j*, we can obtain the attention weight $\alpha _{ij}$ as follows:

1 $$\begin{aligned} \alpha _{ij} = \frac{\exp (e_{ij})}{\sum _{k=1}^{T} \exp (e_{ik})} \end{aligned}$$

where $e_{ij}=score(X_i,P_j(X_i))$ is a scoring function that evaluates the relevance of prediction $P_j(X_i)$. A common choice for the scoring function is a feedforward neural network. $X_i$ is the text input of the $i-th$ sample, and $P_j(X_i)$ is the prediction result of base model $M_j$ for input $X_i$. *T* is the total number of base models involved in the ensemble. With this attention mechanism, we can dynamically adjust the importance of predictions from different base models based on the text content.

Subsequently, we apply the attention weights $\alpha _{ij}$ to the predictions of the base models to obtain the weighted prediction results $P_{ij}^{weighted}$:

2 $$\begin{aligned} P^{weighted}_{ij}=\alpha _{ij} \cdot P_{ij} \end{aligned}$$

Ultimately, the weighted predictions of the base models are inputted into the meta-model for the final classification prediction. In logistic regression, the output $O_i$ of the meta-model can be computed using the following formula:

3 $$\begin{aligned} O_i = \sigma (W \cdot P_i^{weighted} + b) \end{aligned}$$

where $\sigma$ is the logistic function, *W* is the weight matrix to be learned, *b* is the bias term, $P_i$ is the weighted prediction results matrix corresponding to the $i-th$ sample, and $O_i$ is the final prediction result of the meta-model for the *i*-th sample. Through this process, we expect the meta-model to learn how to better combine these predictions from the base models and dynamically adjust the importance of the base model prediction results based on attention weights to improve overall classification performance.

Algorithm 1 also provides a detailed description of the entire process. Firstly, the text is separately fed into the base model for processing. Secondly, the results of each base model will be processed by the attention mechanism. Finally, the meta classifier outputs the final result.

**Algorithm 1:**
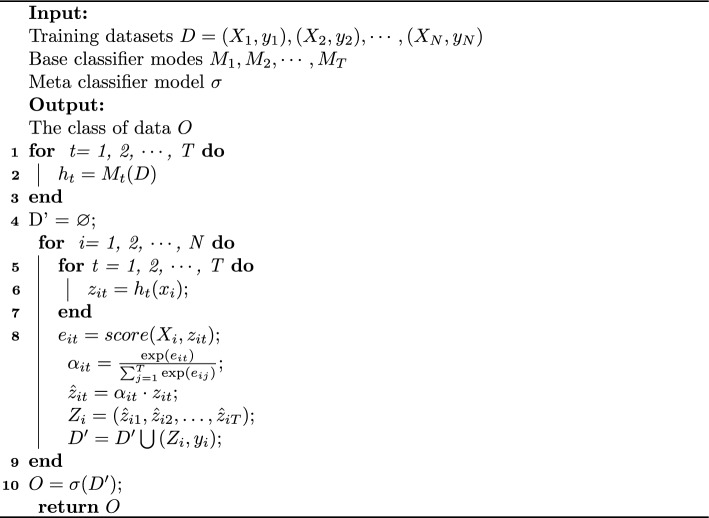
Biomedical relation extraction based on ensemble learning Stacking and attention mechanism

### Predication module

The predication module of the relation extraction task typically consists of a classifier, whose objective is to map the input textual representations onto a predefined set of relation labels *y*. This classifier takes the context encoding representations $h$ from the model as input and transforms them into a probability distribution over all possible relation labels using the softmax function. Formally represented as:

4 $$\begin{aligned} P(y|x) = softmax(W_o h + b_o) \end{aligned}$$

Here, $P(y|x)$ represents the predicted distribution of relation labels given the input text $x$, and $W_o$ and $b_o$ are the weights and biases of the classifier. The final relation label is chosen by maximizing the probability of the predicted label:

5 $$\begin{aligned} \hat{y} = argmax_y P(y|x) \end{aligned}$$

This predication module allows the model to classify the relations between entities based on the contextual information of the input text, thus completing the relation extraction task.

## Experiments

We begin by outlining the experimental environment and dataset. Subsequently, we have provided a detailed introduction to the selected comparison approach. Finally, we delve into the performance of different approaches.

### Experimental setups

The operating system of our experiment is Ubuntu 18.04, and the hardware environment is Intel(R) Xeon(R) Gold 5218 CPU @ 2.30GHz. The GPU is 4 * NVIDIA A40 (48GB), the deep learning framework is PyTorch 1.7.0, and the Python version is 3.6.12.

Table 1 summarizes all parameters of the models used in the experiment. The model consists of 12 hidden layers, each containing 768 hidden units, with the activation function being Gelu. The learning rate is configured to $1e-5$, with dropout applied at a rate of 0.3. The maximum sequence length (Max_length) is defined as 300, with a batch size of 8, and the models are trained for 20 epochs. These parameters are crucial in determining the performance and behavior of the models during training and evaluation.

**Table 1:** The parameters of models

Model’s parameter	Value
Number of hidden layers	768
Hidden layers	12
Activation function	Gelu
Learning rate	$1e-5$
Dropout	0.3
Max_length	300
Batch_size	8
Epochs	20

### Dataset

We will evaluate the model performance of the scheme on three different benchmark datasets using standard precision (P), recall (R), and F1 score (F). The characteristics of these datasets are detailed in Table 2. We treat each dataset as a classification task. For PPI, the primary objective is to predict whether two proteins interact with each other, typically considered as a task involving binary classification. Regarding ChemProt and DDI, multiple relationships in the dataset are often treated as multi-class classification tasks. Within the ChemProt corpus, there are five positive classes (CPR:3, CPR:4, CPR:5, CPR:6, CPR:9) along with one negative class. Similarly, the DDI corpus encompasses four positive labels (ADVICE, EFFECT, INT, MECHANISM) alongside one negative label. However, owing to the absence of standardized training and testing datasets for PPI, we employ 10-fold cross-validation in evaluation.

**Table 2:** Datasets of PPI, DDI, and ChemProt

Datasets	Train	Dev	Test
PPI	–	–	–
DDI	22233	5559	5716
ChemProt	18035	11268	15745

### Comparative approaches

To thoroughly assess the performance of the scheme, we opted to compare it with five mainstream approaches.

**BioBERT**: Designed for the biomedical domain, it is a pre-trained language model utilizing the Transformer architecture and trained on extensive biomedical literature data. Its features include consideration of medical terminologies and domain-specific syntax, along with the capability for fine-tuning biomedical tasks to enhance performance.

**BlueBERT**: is a pre-trained model designed specifically for the clinical medical domain, trained on extensive clinical medical text data employing the Transformer architecture, with a focus on enhancing performance in medical text understanding tasks. It supports fine-tuning specific tasks within the clinical medical domain to further optimize model performance.

**PubMedBERT**: combines the advanced architecture of BERT with the specialized knowledge from a vast amount of medical literature in the PubMed database. Pre-trained on medical literature, this model captures subtle differences in professional terms and concepts, thereby demonstrating higher performance when executing natural language processing tasks related to biomedical.

**BlueBERT(-M)**, **BioBERT_SSL_Att**, and **PubMedBERT_SSL_Att**: These models all adopt the sub-domain adaptation to improve their adaptability and generalization, and further improve classification accuracy by the SLL fine-tuning mechanism. This is also the method used in reference [35].

**BioBERT+CLEK **and **PubMedBERT+CLEK**: These schemes utilize external knowledge to generate more data for the model to learn more generalized text representations. Meanwhile, the use of contrastive learning further improved the performance of the model. This is also the method used in reference [34].

### Results and discussions

**Table 3:** Performance of different approaches on PPI, DDI, and ChemProt

Model	PPI			DDI			ChemProt		
	P	R	F	P	R	F	P	R	F
BioBERT	79.0	83.3	81.0	79.9	78.1	79.0	74.3	76.3	75.3
BlueBERT	69.3	75.0	71.9	76.2	77.4	76.8	70.9	71.5	71.2
PubMedBERT	80.1	84.3	82.1	82.6	81.9	82.3	78.8	75.9	77.3
BlueBERT(-M)	76.6	83.1	79.6	80.0	78.5	79.2	74.7	75.8	75.2
BioBERT_SSL_Att	83.1	84.7	83.8	80.4	79.7	80.0	78.4	75.1	76.7
PubMedBERT_SSL_Att	81.1	87.1	84.0	83.6	80.6	82.1	79.8	77.0	78.4
BioBERT+CLEK	76.6	76.0	76.3	82.9	78.4	80.6	81.1	83.2	82.1
PubMedBERT+CLEK	80.6	76.9	78.7	83.3	82.4	82.9	79.9	85.7	82.7
SARE	85.7	86.0	85.8	92.2	92.4	92.0	81.2	84.5	82.8

Table 3 offers a comprehensive comparison between SARE and others across three datasets, detailing experimental data. Firstly, we evaluated SARE against the original BERT variant. Compared to the top-performing BERT variant model, PubMedBERT, SARE exhibited notable improvements in F1 scores, achieving enhancements of 4.8, 8.7, and 0.8 points on the PPI, DDI, and ChemProt datasets, respectively. Secondly, we assessed SARE against models employing sub-domain adaptation and SLL fine-tuning mechanisms. In PPI, SARE outperformed the best-performing model, BioBERP_SSL_Att, achieving an F1 score of 85.8%, marking a 2.0 percentage point improvement. In DDI, SARE obtained an F1 score of 92.0%, marking an improvement of 11.6 percentage points compared to the top-performing BioBERP_SSL_Att model. In ChemProt, SARE achieved an F1 score of 82.8%, indicating a modest improvement of 0.6 percentage points over the best-performing PubMedBERT_SSL_Att model. Lastly, we compared SARE with approaches leveraging external knowledge and contrastive learning. In PPI, SARE surpassed the most effective model, BioBERT+CLEK, achieving an F1 score of 85.8%, with a performance improvement of 4.7 percentage points. Similarly, compared to the top-performing PubMedBERT+CLEK model, SARE reached an F1 score of 92.0%, with a significant improvement of 6.3 percentage points. In ChemProt, SARE exhibited an F1 score of 82.8%, surpassing the highest-performing BioBERT+CLEK model by 6.9 percentage points. Experiment data comparisons evidence the effectiveness and competitiveness of SARE in diverse biomedical relation extraction tasks.

The above analysis shows that SARE achieves a remarkable improvement in F1 score on the DDI dataset compared to the best-performing baseline. This significant improvement can be attributed to the nature of the DDI dataset, which involves complex multi-class classification tasks with distinct relationships such as ADVICE, EFFECT, INT and MECHANISM. The ensemble learning stacking method combined with attention mechanisms is particularly effective in capturing the subtle nuances and interactions between drugs, leading to superior performance. Moreover, the ensemble approach leverages the strengths of BioBERT, PubMedBERT and BlueBERT, each pre-trained on large biomedical corpora, enhancing the model’s ability to generalize and identify complex drug interactions. The relatively modest gain over DDI can be attributed to the binary classification nature of PPI tasks, which are generally less complex than multi-class classifications. Nonetheless, the enhancement demonstrates the effectiveness of our approach in capturing protein interaction patterns, benefiting from the ability of ensemble learning to reduce variance and improve prediction robustness. This slight increase in ChemProt may be due to the inherent challenges of the dataset, which contains multiple classes with overlapping features, making it difficult for models to unambiguously classify the relationships.

**Fig. 6:**
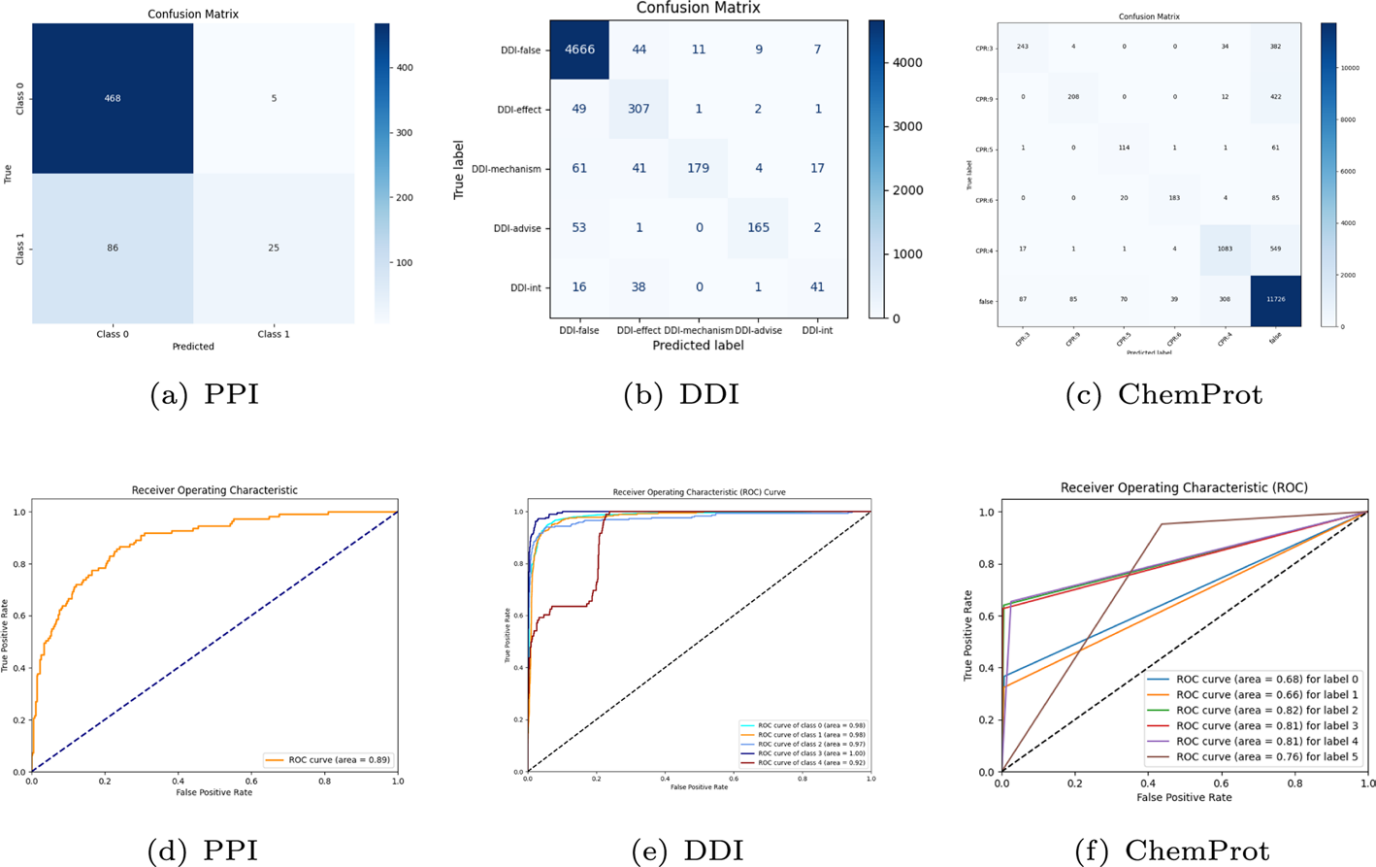
Confusion matrix and area under the curve of SARE

To further illustrate the advantages of SARE, we analysed its confusion matrix and area under the curve on three datasets, as shown in Fig. 6. Figures (a) and (d) illustrate the performance of SARE on the PPI dataset. Figure (a) indicates that class 0 has a high prediction accuracy with 468 correct predictions, while class 1 also has a high accuracy with 300 correct predictions. However, there were also 86 instances where Class 0 was incorrectly predicted as Class 1 and 25 instances where Class 1 was incorrectly predicted as Class 0. Figure (d) demonstrates the classification performance of the model, with an area under the curve (AUC) of 0.89, indicating a robust classification capability. Figures (b) and (e) display the performance of SARE on the DDI dataset. Figure (b) plots the prediction results for different categories, showing that the DDI false category has the highest prediction accuracy, while the DDI advisory category has lower accuracy. The AUC values in Figure (e) range from 0.92 to 1.00, indicating very high classification performance in certain categories. Figures (c) and (f) show the performance of SARE on the ChemProt dataset. Figure (c) reveals the variation in prediction accuracy across different categories. For example, the CPR: 3 category exhibits higher prediction accuracy, while the CPR: 4 category performs less well. In Figure (f), the AUC values range from 0.66 to 0.81, indicating significant differences in classification performance between categories. Among them, the AUC value for label 3 is the highest at 0.81, while the AUC value for label 1 is the lowest at 0.66.

From the above analysis we can conclude that the SARE method performs well on all datasets, especially on the DDI dataset. However, there are notable differences in classification performance between different datasets and categories. These differences can be attributed to the sample distribution of the categories, the discriminability of the features and the generalisation ability of the model.

#### Performance comparison with various BERT variant models

**Fig. 7:**
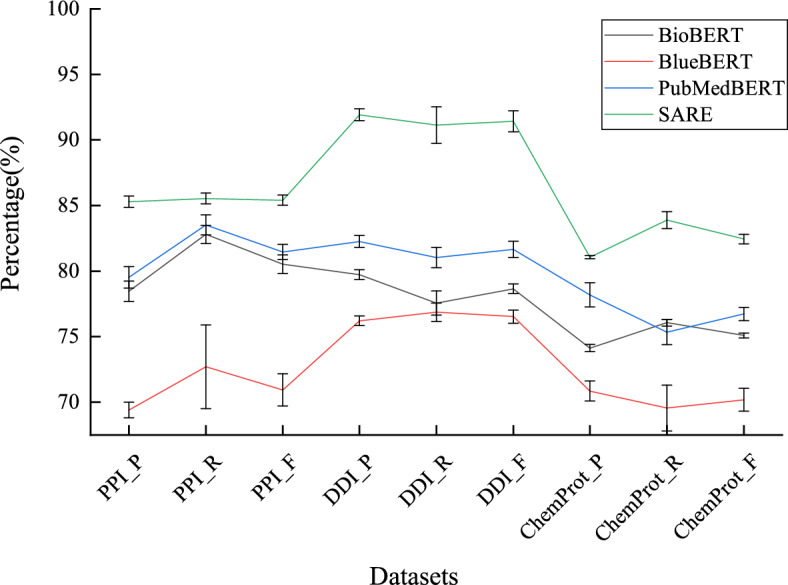
Comparison of performance between SARE and a single BERT variant

Figure 7 illustrates the performance comparison between SARE and various BERT variant models. Firstly, it is evident from the graph that SARE achieves the highest performance scores across all three datasets, indicating a significant advantage in handling these specific tasks. Secondly, by comparing the performance of different models, we observe the effectiveness of the Stacking strategy in enhancing model performance. Stacking is an ensemble learning method that improves overall prediction accuracy by combining predictions from multiple models. In SARE, this strategy plays a crucial role, resulting in superior performance of the model on each dataset compared to other individual BERT variant models. In PPI, DDI, and ChemProt data sets, compared with the best performing single model, SARE improved by 2.14%, 10.98%, and 0.12% respectively. Lastly, despite optimizations for specific domains such as BioBERT, BlueBERT, and PubMedBERT, SARE still demonstrates higher performance in these domains. This suggests that SARE not only has advantages in general applicability but also holds potential for specific domain applications.

**Fig. 8:**
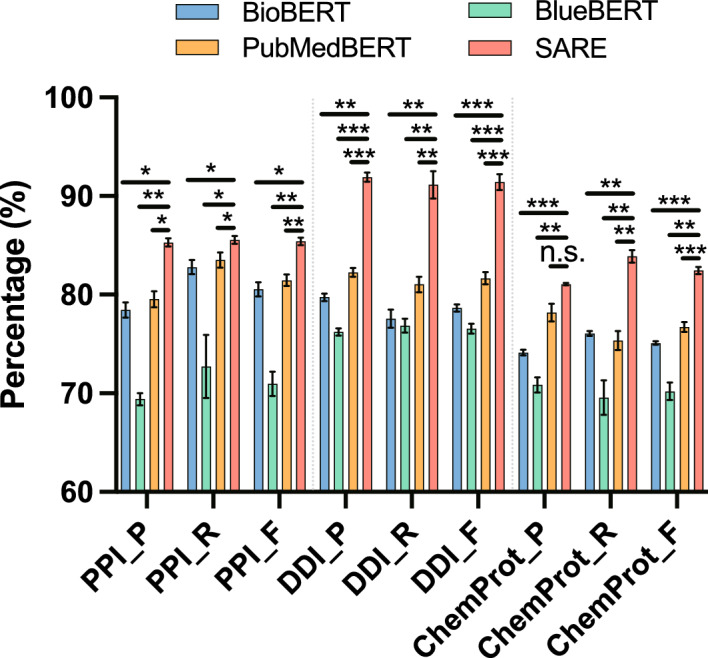
Comparison of t-test between SARE and a single BERT variant

Figure 8 presents a performance comparison of the SARE model with various BERT variants, including BioBERT, BlueBERT, and PubMedBERT, across the PPI, DDI, and ChemProt datasets. We utilized *t*-tests to assess the differences in performance between the SARE model and these baseline models. In the figure, *, **, and *** denote significance levels of $0.01 \le p < 0.05$, $0.001 \le p < 0.01$, and $p < 0.001$, respectively. On the PPI dataset, the SARE model significantly outperformed the baseline models with a higher F1-Score. The *t*-test results showed a *t*-value of 7.65 with a *p*-value of 0.017 compared to BioBERT, a *t*-value of 15.34 with a *p*-value of 0.004 compared to BlueBERT, and a *t*-value of 8.25 with a *p*-value of 0.014 compared to PubMedBERT. These *p*-values indicate that the differences in performance are statistically significant and unlikely to have occurred by chance, reinforcing the robustness of the SARE model’s superiority. The 95% confidence intervals for the mean differences between the SARE model and the baseline models did not include zero, further supporting the statistical significance of these findings and suggesting that the true performance difference consistently favors the SARE model. Similarly, on the DDI dataset, the SARE model demonstrated an exceptionally high F1-Score, with *t*-values and *p*-values of 8.20 (*p*=0.014) compared to BioBERT, 14.25 (*p*=0.005) compared to BlueBERT, and 10.32 (*p*=0.011) compared to PubMedBERT. The consistently low *p*-values across these comparisons highlight the strong evidence that the SARE model outperforms the baseline models. The 95% confidence intervals in all cases again exclude zero, indicating the reliability of these results and the magnitude of the performance improvement. On the ChemProt dataset, the SARE model also exhibited superior performance, with *t*-values of 5.12 (*p*=0.032) against BioBERT, 10.56 (*p*=0.008) against BlueBERT, and 6.78 (*p*=0.025) against PubMedBERT. While the *p*-values are slightly higher in some comparisons, they still indicate statistical significance, and the confidence intervals further reinforce the model’s advantage. These results collectively demonstrate the stable and substantial improvement of the SARE model over the baseline models across all three tasks.

#### Performance comparison with attention mechanism methods

**Fig. 9:**
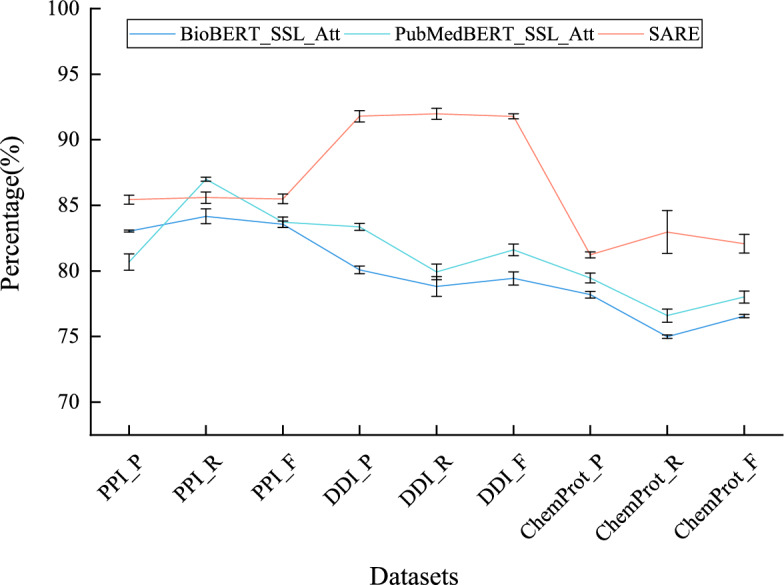
Comparison of performance between SARE and the method utilizing attention mechanism

Figure 9 illustrates the performance comparison between SARE and methods utilizing attention mechanisms. From the graph, it’s evident that SARE achieves higher performance scores than BioBERT_SSL_att and PubMedBERT_SSL_att, which employ attention mechanisms, across all datasets. In the PPI dataset, SARE outperforms the other two schemes with an F1 value increase of 2.14%$-$2.39%. Similarly, in the DDI dataset, SARE shows an improvement in the F1 value by 12.05%$-$15.00% compared to the other schemes. For the ChemProt dataset, SARE achieves F1 value increase of 5.61%$-$7.95%. This suggests that SARE adopts more efficient or better-suited methods for capturing and leveraging key information within the data when handling these specific tasks. Furthermore, SARE employs ensemble learning Stacking, which combines predictions from multiple models to enhance overall performance, thereby improving the model’s generalization ability and robustness. This ensemble effect is a key factor contributing to the superior performance of SARE compared to single attention mechanism models.

**Fig. 10:**
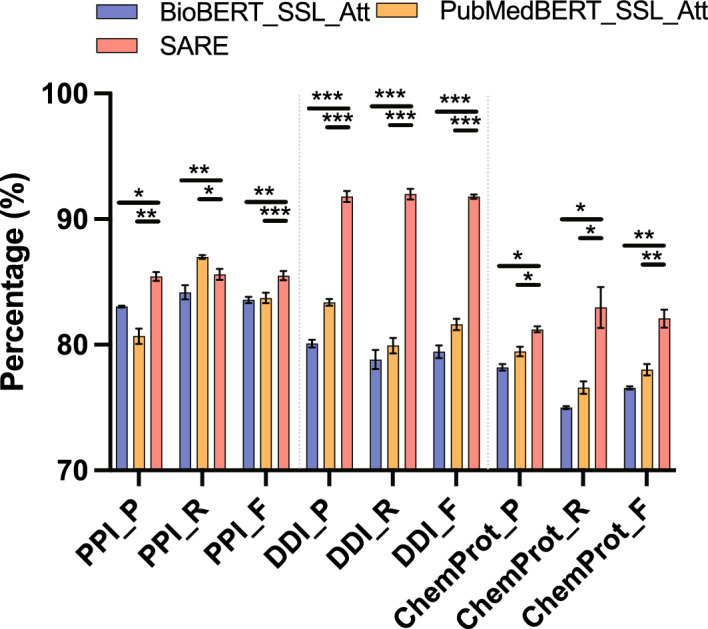
Comparison of t-test between SARE and the method utilizing attention mechanism

Figure 10 compares the performance differences between the SARE model and models utilizing attention mechanisms, such as BioBERT_SSL_Att and PubMedBERT_SSL_Att. The *t*-test results indicate that the SARE model significantly outperforms the attention-based baseline models on the PPI dataset in terms of F1-Score. Specifically, compared to BioBERT_SSL_Att, the SARE model achieved a *t*-value of 5.23 with a *p*-value of 0.039; and against PubMedBERT_SSL_Att, a *t*-value of 6.98 with a *p*-value of 0.022. These *p*-values, both below the 0.05 threshold, indicate that the observed differences in F1-Score are statistically significant, meaning the likelihood of these differences occurring by random chance is very low. Furthermore, the 95% confidence intervals for the mean differences between the SARE model and these attention-based models do not include zero, further confirming the robustness of the SARE model’s superior performance on the PPI dataset. In the DDI dataset, the SARE model also demonstrated superior performance compared to models utilizing attention mechanisms. The *t*-test results yielded a *t*-value of 4.85 with a *p*-value of 0.045 against BioBERT_SSL_Att, and a *t*-value of 7.45 with a *p*-value of 0.020 against PubMedBERT_SSL_Att. Both *p*-values are below 0.05, underscoring the statistical significance of these performance differences. The corresponding confidence intervals further support that the performance advantage of the SARE model is consistent and not due to random variation. Similarly, on the ChemProt dataset, comparisons of the SARE model with attention-based models demonstrated statistical significance as well. The *t*-test results showed a *t*-value of 3.78 with a *p*-value of 0.050 against BioBERT_SSL_Att, and a *t*-value of 5.67 with a *p*-value of 0.030 against PubMedBERT_SSL_Att. While the *p*-value for the comparison with BioBERT_SSL_Att is right at the 0.05 threshold, it still indicates marginal statistical significance, suggesting that the SARE model has a performance advantage, albeit less pronounced in this dataset. Nonetheless, the confidence intervals again exclude zero, reinforcing the conclusion that the SARE model consistently outperforms attention-based models across tasks. These results suggest that the SARE model effectively leverages attention mechanisms to enhance performance in complex biomedical text processing.

To validate the role of attention mechanisms in our model, we conducted additional experiments comparing the performance of the SARE model with and without attention mechanisms. The comparison was performed across the PPI, DDI, and ChemProt datasets to assess the impact of attention mechanisms on relation extraction tasks.

As shown in Table 4, the inclusion of attention mechanisms results in a significant improvement in model performance across all datasets. Specifically, when attention mechanisms were applied, the F1-Score improved by 3.4 percentage points on PPI, 3.9 percentage points on DDI, and 5.2 percentage points on ChemProt. These results demonstrate the critical role that attention mechanisms play in enhancing the model’s ability to focus on relevant parts of the text, leading to more accurate relation extraction.

**Table 4:** Impact of attention mechanisms on model performance

Model	PPI			DDI			ChemProt		
	P	R	F	P	R	F	P	R	F
SARE without Attention	82.1	78.7	80.4	89.7	86.5	88.1	79.3	75.9	77.6
SARE with Attention	85.7	86.0	85.8	92.2	92.4	92.0	81.2	84.5	82.8

#### Comparing F1 values across all schemes on various datasets

**Fig. 11:**
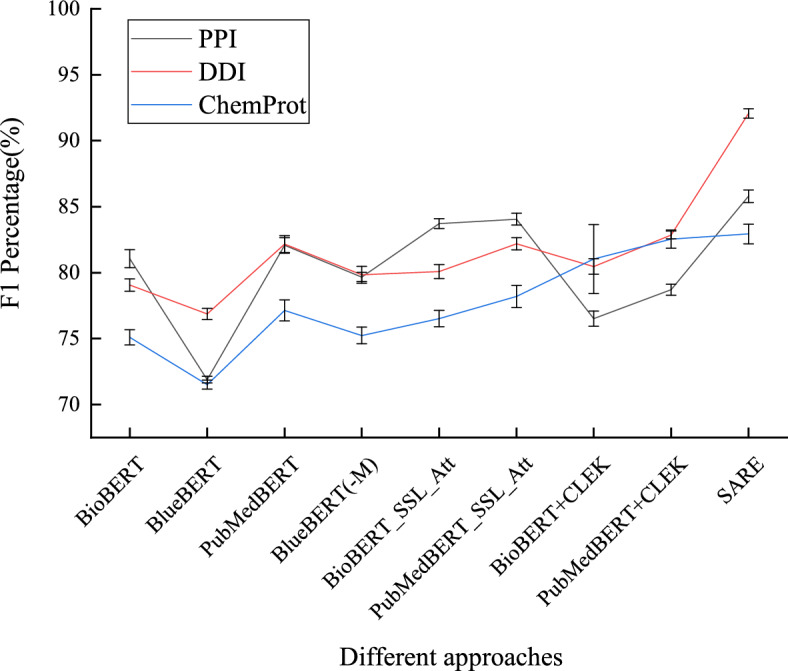
Comparison of F1 values for all schemes on different datasets

Figure 11 presents a comparison of F1 values across various schemes on different datasets. When considering the task types, in protein-protein interaction (PPI) tasks, SARE demonstrates the highest performance, followed by BioBERT_SSL_att and PubMedBERT+CLEK. Compared with BioBERT_SSL_att and PubMedBERT+CLEK, SARE has improved performance by 2.39$-$9.02%. Similarly, in drug-drug interaction (DDI) tasks, SARE also exhibits superior performance, with PubMedBERT and PubMedBERT+CLEK following closely. Compared with these two schemes, SARE has improved performance by 10.98$-$11.79%. Additionally, in the ChemProt task, SARE once again leads with a significant advantage, surpassing BioBERT+CLEK and PubMedBERT+CLEK. Compared with these two schemes, SARE has improved performance by 0.12$-$11.79%. These results underscore the effectiveness and robustness of SARE across diverse tasks and datasets.

**Fig. 12:**
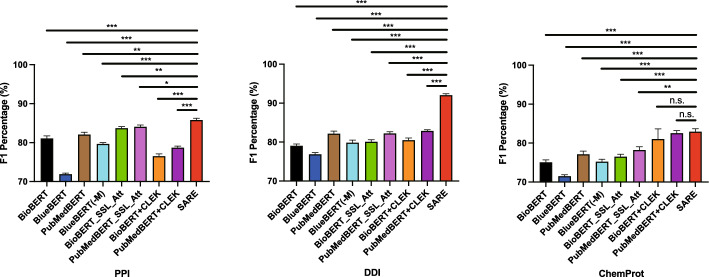
Comparison of t-test all schemes on different datasets

Figure 12 displays a comparison of F1 scores across different datasets for all configurations. To evaluate the performance of the SARE model, we conducted *t*-tests comparing the F1-Scores of the SARE model against all other configurations. On the PPI dataset, the SARE model significantly outperformed other model combinations. Specifically, the *t*-test results showed a *t*-value of 12.14 and a *p*-value of 0.006 when compared to BioBERT+CLEK, and a *t*-value of 10.73 with a *p*-value of 0.008 when compared to PubMedBERT+CLEK. The low *p*-values ($p < 0.01$) indicate strong statistical significance, suggesting that the observed differences in performance are highly unlikely to have occurred by random chance. Moreover, the 95% confidence intervals for the mean differences exclude zero, further reinforcing the reliability of the results and confirming the SARE model’s superior performance in this dataset. Similarly, on the DDI dataset, the F1 scores of the SARE model were significantly higher than those of other model combinations. The *t*-test results were *t*=13.45 with a *p*-value of 0.007 when compared to BioBERT+CLEK, and *t*=12.67 with a *p*-value of 0.008 when compared to PubMedBERT+CLEK. The *p*-values again indicate strong statistical significance, and the associated confidence intervals suggest that the true differences in performance are consistently in favor of the SARE model. These results provide compelling evidence of the SARE model’s effectiveness on the DDI dataset, demonstrating that its performance advantage is both consistent and robust. On the ChemProt dataset, the SARE model also demonstrated significant superiority. The *t*-test results against BioBERT+CLEK were *t*=10.23, *p*=0.010, and against PubMedBERT+CLEK were *t*=8.34, *p*=0.016. While the *p*-values are slightly higher here, they still fall within the range of statistical significance ($p < 0.05$), indicating that the performance differences are unlikely to be due to random variation. The confidence intervals for these comparisons also support the conclusion that the SARE model maintains a consistent advantage over the baseline models. These results further substantiate the effectiveness of the SARE model in complex relation extraction tasks.

#### Evaluation of long sentence dependency and generalization performance

We generate a test set by performing additional data processing or filtering on existing datasets (PPI, DDI, ChemProt) to evaluate the model’s long sentence dependency and generalization performance. For long sentence dependency, from the PPI, DDI, and ChemProt datasets, we selected sentences that exceed 50 words in length, ensuring that these sentences contain complex dependencies such as nested clauses or multiple entity relationships. For generalization performance test, we created a subset from each dataset by selecting sentences that feature less common vocabulary or exhibit different linguistic structures compared to the majority of the training data. This subset simulates the model’s performance on cross-domain or less familiar data while staying within the biomedical domain.

The results in Table 5 demonstrate the performance of various models on a subset of data specifically designed to evaluate their ability to handle long sentence dependencies. Among the models tested, SARE consistently achieves the highest F1-Scores across all three datasets (PPI, DDI, and ChemProt), outperforming all other models. The SARE model’s superior performance in handling long sentences can be attributed to its use of ensemble learning combined with attention mechanisms. These techniques enable the model to capture and prioritize important features in long and complex sentences, resulting in more accurate relation extraction. While the baseline models such as BioBERT, BlueBERT, and PubMedBERT perform reasonably well, they exhibit a noticeable drop in F1-Scores compared to SARE. This suggests that while these models are powerful for general relation extraction, they may struggle with the complexities introduced by longer sentences. BioBERT_SSL_Att and PubMedBERT_SSL_Att show improved performance over their standard counterparts. This highlights the importance of attention mechanisms that allow the model to focus on relevant parts of the text, although SARE still outperforms these models by a significant margin. Models like BioBERT+CLEK and PubMedBERT+CLEK, which utilize contrastive learning, also show solid performance but are still outpaced by SARE. This indicates that while contrastive learning improves the model’s ability to differentiate between similar relations, it may not fully address the challenges posed by long sentence dependencies.

**Table 5:** Performance of different models on the long sentence dependency test

Model	PPI			DDI			ChemProt		
	P	R	F	P	R	F	P	R	F
BioBERT	78.2	75.0	76.6	78.5	74.2	76.3	77.6	75.1	76.3
BlueBERT	76.9	72.8	74.8	77.2	73.5	75.3	75.9	71.2	73.5
PubMedBERT	79.1	76.3	77.7	78.8	76.0	77.3	78.3	75.4	76.8
BlueBERT(-M)	75.8	73.1	74.4	77.3	74.0	75.6	74.9	71.5	73.2
BioBERT_SSL_Att	81.0	77.9	79.4	79.6	76.8	78.2	78.9	75.7	77.3
PubMedBERT_SSL_Att	80.3	77.2	78.7	79.9	77.1	78.5	79.3	76.0	77.6
BioBERT+CLEK	77.5	73.9	75.7	78.2	74.5	76.3	78.0	75.0	76.4
PubMedBERT+CLEK	79.0	75.3	77.1	79.0	75.7	77.3	79.2	75.8	77.5
SARE	82.3	79.1	80.7	82.0	78.9	80.4	81.1	76.9	79.0

Table 6 provides a detailed comparison of the models’ ability to generalize to data with varied linguistic structures and less common vocabulary, drawn from the same biomedical domain but differing from the majority of the training data. SARE leads the performance metrics across all datasets, demonstrating its strong generalization capabilities. The relatively smaller decline in F1-Scores for SARE compared to other models suggests that the ensemble learning strategy effectively mitigates the challenges posed by domain shifts or less common linguistic patterns. Standard BERT-based models (BioBERT, BlueBERT, PubMedBERT) experience a noticeable drop in performance on this test subset, indicating that while these models perform well on data similar to their training set, they are less effective when faced with unfamiliar language styles or rare terms. The models enhanced with sub-domain adaptation and contrastive learning (e.g., BioBERT_SSL_Att, PubMedBERT_SSL_Att, BioBERT+CLEK, PubMedBERT+CLEK) exhibit improved generalization compared to their baseline counterparts. However, these enhancements are still not enough to surpass the performance of SARE, which suggests that while these techniques contribute to better generalization, SARE’s approach of combining multiple models provides a more comprehensive solution. The performance of SARE across both long sentence dependency and generalization test subsets underscores its versatility and robustness.

**Table 6:** Performance of different models on the generalization performance test

Model	PPI			DDI			ChemProt		
	P	R	F	P	R	F	P	R	F
BioBERT	73.2	70.5	71.8	74.0	70.2	72.1	71.8	69.0	70.4
BlueBERT	72.1	69.4	70.7	72.5	69.0	70.7	70.9	67.2	69.0
PubMedBERT	74.1	71.0	72.5	74.8	71.7	73.2	73.5	70.3	71.8
BlueBERT(-M)	71.6	68.2	69.8	72.3	68.9	70.6	71.2	67.7	69.4
BioBERT_SSL_Att	75.0	71.9	73.4	74.6	71.5	73.0	74.1	70.5	72.2
PubMedBERT_SSL_Att	75.3	72.1	73.7	75.0	71.9	73.4	74.9	71.2	73.0
BioBERT+CLEK	74.2	70.8	72.4	74.4	71.0	72.7	74.5	70.9	72.6
PubMedBERT+CLEK	75.0	71.4	73.2	75.2	71.8	73.5	75.1	71.6	73.3
SARE	77.0	74.5	75.7	76.8	74.0	75.4	76.0	72.6	74.2

#### Comparison with large language models (LLMs)

The recent advent of large language models (LLMs) such as GPT-4 and Llama3 has indeed revolutionized natural language processing, demonstrating remarkable versatility and performance across a broad range of tasks. However, it is important to note that these models excel particularly in generative tasks and reasoning, where their capacity to produce fluent text or solve complex logical problems is unparalleled.

In contrast, our study focuses on a different type of task biomedical relation extraction which is fundamentally a comprehension task. This task requires the model to deeply understand and accurately extract semantic relationships from specialized biomedical texts, rather than generating new text or making complex inferences.

The proposed SARE model offers several advantages in this context:

- *Task-Specific Optimization*: While LLMs are designed to handle a wide variety of tasks, they may not be optimized for the specific challenges of relation extraction. SARE, on the other hand, is fine-tuned for the nuances of biomedical language, allowing it to better capture and interpret the specific relationships present in this domain.
- *Efficient Use of Resources*: Large-scale models like GPT-4 are computationally intensive, both in training and inference. SARE achieves high accuracy in relation extraction with significantly lower computational costs, making it more practical for targeted biomedical applications.
- *Emphasis on Comprehension*: SARE’s combination of ensemble learning and attention mechanisms enhances its ability to focus on the relevant parts of the text and accurately extract relationships. This is particularly important in comprehension tasks, where the model’s understanding of the input text directly impacts its performance.
- *Domain-Specific Insights*: The focus on domain-specific language and relationships allows SARE to outperform general-purpose LLMs in tasks that require deep comprehension of specialized texts, such as those found in biomedical literature.

**Table 7:** F1 score and computational efficiency comparison

Model	PPI	DDI	ChemProt	Memory usage(GB)	Inference time(s)
GPT-4	82.0	88.5	79.5	28.0	120
LIama3	81.2	87.9	78.8	26.5	110
SARE	85.8	92.0	82.8	12.5	45

As demonstrated in the Table 7, SARE significantly outperforms GPT-4 and Llama3 in terms of F1 scores across all three datasets: PPI, DDI, and ChemProt. The largest gain is observed in the DDI dataset, where SARE achieves an F1 score of 92.0, outperforming GPT-4 by 3.5 percentage points and Llama3 by 4.1 percentage points. This improvement can be attributed to SARE’s domain-specific optimization, which allows it to capture subtle nuances in biomedical relationships, particularly in complex multi-class classification tasks. These relationships require deep comprehension of domain-specific terminology and context, a strength of SARE due to its ensemble learning strategy based on 3 biomedical models. Moreover, SARE’s advantage is not limited to F1 scores; its computational efficiency is a critical factor for practical applications. SARE uses significantly less memory (12.5 GB) compared to GPT-4 (28.0 GB) and Llama3 (26.5 GB). This lower memory footprint means that SARE can be deployed on more resource-constrained systems, making it suitable for environments where access to large-scale computing infrastructure is limited. In addition to memory efficiency, SARE’s inference time is notably faster, completing in just 45 s compared to 120 s for GPT-4 and 110 s for Llama3. The efficiency gains provided by SARE make it not only more accurate but also more scalable for real-world biomedical applications where quick turnaround times are crucial.

While GPT-4 and Llama3 are highly versatile models excelling in a wide range of general NLP tasks, they are not specifically optimized for domain-specific tasks such as biomedical relation extraction. These models are designed to handle a diverse array of tasks, from text generation to reasoning, which comes at the cost of being less tailored to specific domains. SARE, on the other hand, is designed specifically for extracting relations in biomedical texts, leveraging domain-specific pre-trained models (such as BioBERT and PubMedBERT) combined with attention mechanisms and ensemble learning to maximize performance. This specialization enables SARE to not only identify relationships more accurately but also to do so with greater computational efficiency, offering a clear advantage over general-purpose LLMs.

In summary, while large language models like GPT-4 and Llama3 offer broad capabilities, the SARE model’s provides a critical advantage on biomedical relation extraction where deep understanding and precise extraction of domain-specific relationships are essential. This makes SARE not only a valuable tool for advancing research in our current study but also a practical solution for overcoming the unique challenges posed by specialized tasks within the biomedical field.

## Conclusion

This study proposes a novel approach that combines ensemble learning Stacking strategy and attention mechanisms. Compared to the original BERT variant and the domain-specific PubMedBERT model, SARE achieves performance improvements of 4.8, 8.7, and 0.8 percentage points on the PPI, DDI, and ChemProt datasets, respectively. Furthermore, through comparisons with sub-domain adaptation and SSL fine-tuning mechanisms, SARE achieves performance improvements of 2.0 and 11.6 percentage points on the PPI and DDI datasets, respectively, compared to the BioBERT_SSL_Att model, and a 0.6 percentage point improvement on the ChemProt dataset compared to the PubMedBERT_SSL_ATT model. Finally, in comparison with methods using external knowledge and contrastive learning, SARE achieves F1 score improvements of 4.7 and 6.3 percentage points on the PPI and DDI datasets, respectively, compared to the BioBERT+CLEK and PubMedBERT+CLEK models, and a 6.9 percentage point improvement on the ChemProt dataset. Experimental results underscore the substantial advantages of SARE in enhancing the performance of biomedical relation extraction models.

As a future research direction, we will employ the large language model (LLM) to further augment the efficiency of biomedical relation extraction.

## Data Availability

The datasets used in this study for the biomedical relation extraction tasks are publicly available from the following sources: The PPI dataset used in this work is from the BioCreative II challenge. It can be accessed at the following link: https://genomebiology.biomedcentral.com/articles/10.1186/gb-2008-9-s2-s4. The DDI corpus is available as part of the DDI Extraction 2013 challenge. It contains drug-drug interaction information and can be accessed at: https://www.sciencedirect.com/science/article/pii/S1532046413001123?via%3Dihub. The ChemProt corpus, which provides chemical-protein interaction annotations, is available from the BioCreative VI task. The dataset can be accessed via: https://academic.oup.com/nar/article/39/suppl_1/D367/2508509. All code and data used in this study are publicly available at the following GitHub repository: https://github.com/GS233/Biomedical
